# Nitrite binding to globins: linkage isomerism, EPR silence and reductive chemistry

**DOI:** 10.1016/j.niox.2014.08.007

**Published:** 2014-11-15

**Authors:** Radu Silaghi-Dumitrescu, Dimitri A. Svistunenko, Daniela Cioloboc, Cristina Bischin, Florina Scurtu, Chris E. Cooper

**Affiliations:** a “Babeş-Bolyai” University, 1 Mihail Kogalniceanu str., RO-400084 Cluj-Napoca, Romania; b Department of Biological Sciences, University of Essex, Wivenhoe Park, Colchester, Essex CO4 3SQ, UK

**Keywords:** Hemoglobin, Myoglobin, Nitrite, DFT, EPR

## Abstract

The nitrite adducts of globins can potentially bind via O- or N- linkage to the heme iron. We have used EPR (electron paramagnetic resonance) and DFT (density functional theory) to explore these binding modes to myoglobin and hemoglobin. We demonstrate that the nitrite adducts of both globins have detectable EPR signals; we provide an explanation for the difficulty in detecting these EPR features, based on uniaxial state considerations. The EPR and DFT data show that both nitrite linkage isomers can be present at the same time and that the two isomers are readily interconvertible in solution. The millisecond-scale process of nitrite reduction by Hb is investigated in search of the elusive Fe(II)-nitrite adduct.

## Highlights

• A DFT-derived barrier for nitrite linkage isomerism on heme center is reported.
• EPR spectra of nitrite adducts show evidence for linkage isomerism.
• The electronic structure of Fe(III)-nitrite heme is conformation-dependent.
• Certain conformations are inducive to EPR silence.
• Fe(II)-nitrite is undetectable on stopped-flow time scales.

## Graphic Abstract

Graphic entry for the Table of Contents (TOC).

**Figure:**
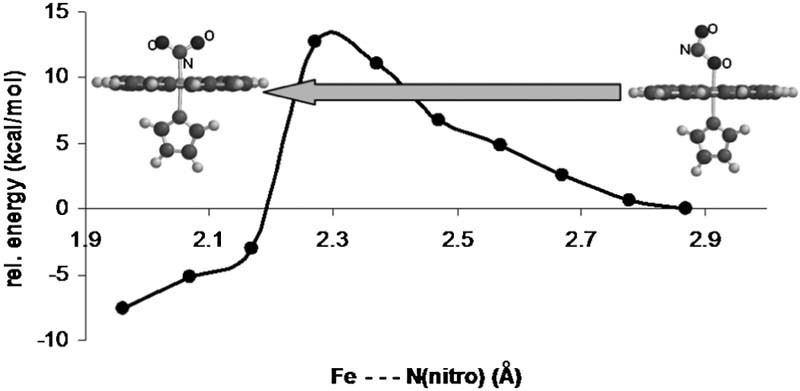

## 1 Introduction

**Fig. 1:**
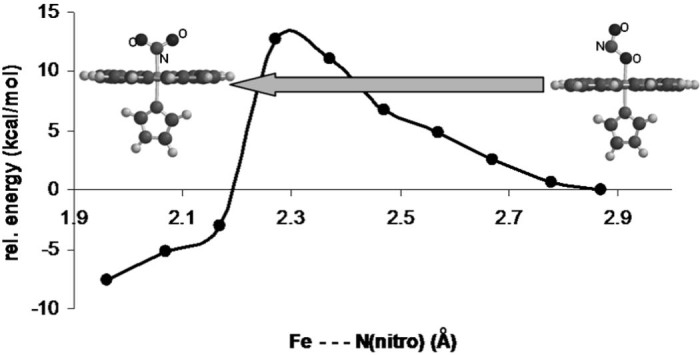
Potential energy surface for nitrite linkage isomerization on a model of ferric myo/hemoglobin (UBP86/6-31G**).

Nitrite binds to the reduced and oxidized forms of heme proteins and, in enzymes such as cytochrome cd_1_ nitrite reductase and cytochrome c nitrite reductase, undergoes reduction to nitric oxide or ammonia [1], [2], [3], [4], [5], [6], [7], [8], [9]. More recently, interest has been focused upon nitrite reduction by hemoglobin (Hb), a reaction which has been proposed to have medical/physiological relevance [10], [11], [12], [13], [14], [15], [16]. Nitrite has traditionally been observed to bind to the iron in hemes and hemoproteins via the nitrogen atom (leftmost structure in Fig. 1) [2]. Based on density functional theory (DFT) calculation, we have previously proposed that binding of nitrite to the heme iron via its oxygen atom should also be feasible (rightmost structure in Fig. 1) – hence a nitrite linkage isomerism phenomenon; we further argued that this previously ignored binding mode would have mechanistic and physiological relevance [2]. This hypothesis has since been partially confirmed experimentally by the crystal structures of myoglobin and hemoglobin with nitrite, which have identified nitrite bound to iron *only via the oxygen atom* [17], [18], [19]. From an experimental point of view, this finding places globins (only binding nitrite via oxygen) [18] and cytochrome reductases (only binding nitrite via nitrogen) [1] *in contrast to each other*; notably, the two linkage isomers have not been observed at the same time, in the same reaction mixture, with the same protein. Somewhat related to this issue, EPR spectra have recently been reported for ferric Hb-nitrite adducts, surprisingly showing a complete lack of signals attributable to nitrite-bound Hb [10]. This finding was reminiscent of the similar EPR silence of a nitrite adduct of ferric heme d_1_-nitrite adduct of the enzyme cytochrome cd_1_ nitrite reductase [4], [20]. These EPR silence phenomena have received several possible explanations, none of which are to our knowledge generally accepted. Among these would be a significant degree of structural inhomogeneity, fast rotation of the nitrite ligand around the iron–nitrogen bond, or a so-called uniaxial state as detailed below [10], [18], [20]. On the other hand Singel and co-workers as well as Young and Siegel have reported that nitrite-hemoglobin adducts do show entirely detectable EPR signals, characterized by specific g-values at ~2.9 [20], [21], [22], while others have further shown that such signals show buffer dependence and can indeed appear almost non-detectable under certain conditions [15], [23].

**Fig. 2:**
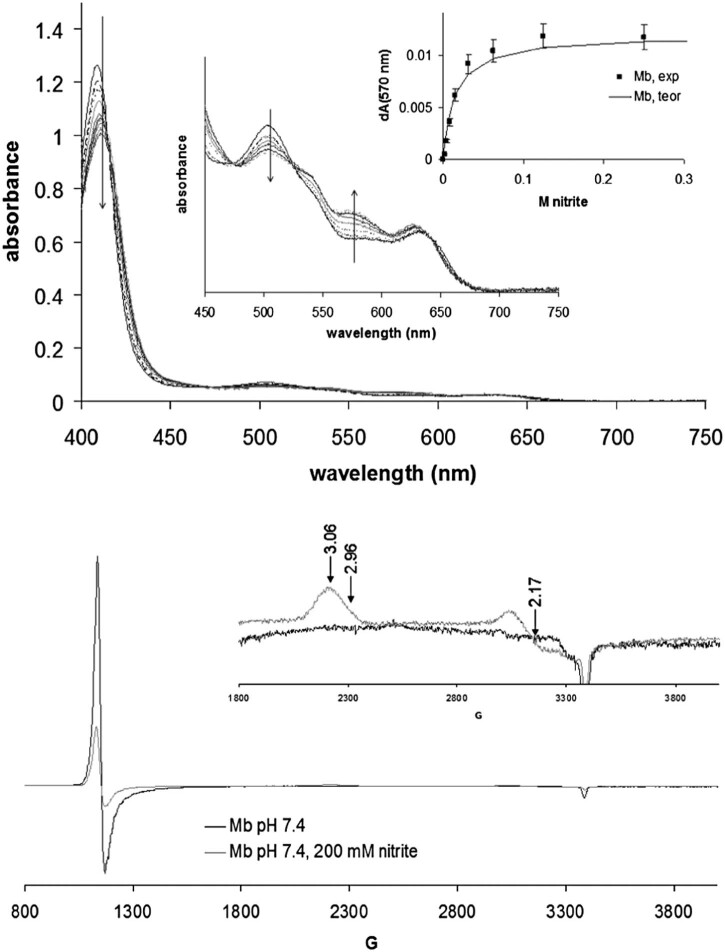
Top panel: UV-vis spectra of ferric myoglobin (100 µM) in the presence of varying amounts of nitrite, in 50 mM phosphate pH 7.4, room temperature; shown as inset is a binding curve whose theoretical fit indicates a binding constant of 14 mM. Bottom panel: EPR spectra of Mb in the presence and absence of nitrite, at 10 K. The g-values of the signals are indicated. Instrument conditions: microwave frequency 9.47 GHz, microwave power 3.18 mW, modulation frequency 100 kHz, modulation amplitude 5 G, sweep rated 22.6 G/s; time constant 81.92 ms, single sweep for each spectrum.

Here, EPR and DFT data are shown on the ferric nitrite adducts of myoglobin and hemoglobin suggesting that indeed both nitrite linkage isomers can be present at the same time and in the same solution in both globins, and that the two isomers can easily interconvert. Straightforward arguments will also be provided to support a previously formulated explanation for the relative EPR silence of heme nitrite adducts.

## 2 Experimental

Hemoglobin was purified following a variation of the general protocol of Antonini and Brunori [24]. Blood (regardless of source – human, bovine, ovine, etc.), freshly drawn on citrate, was centrifuged 15 minutes at 5000 rpm (*g*) to separate the red blood cells, which were then washed three times with 5 mM phosphate pH 7.4 + 150 mM NaCl. Hemoglobin concentrations in text are given per heme rather than per tetramer. Myoglobin (lyophilized, from horse heart) were purchased from Sigma and used without any further purification. The met forms of hemoglobin and myoglobin were prepared by ferricyanide treatment as previously described [25], [26], [27].

Stock solutions (25 mM) of DEA NONOate (diethylammonium (Z)-1-(N,N-diethylamino) diazen-1-ium-1,2–40 diolate, Cayman Chemicals, Inc.) were prepared in 0.01 M NaOH. DEA NONOate is stable at high pH but decomposes to release NO gas (1.5 mol NO/1 mol DEA NONOate) when added to assay mixtures at ~ pH 7.

Deoxy forms were obtained either by addition of a few grains of dithionite (large excess) to oxy hemoglobin, or by titration with dithionite of globin solutions previously degassed by purging with argon the headspace of rubber-septum-sealed UV-vis cuvettes.

In cases where dithionite was not used the anaerobicity was ensured by utilizing a glucose (10 mM), glucose oxidase (10 Units) and catalase (150 Units) dioxygen-scrubbing system in the assay mix.

**Fig. 3:**
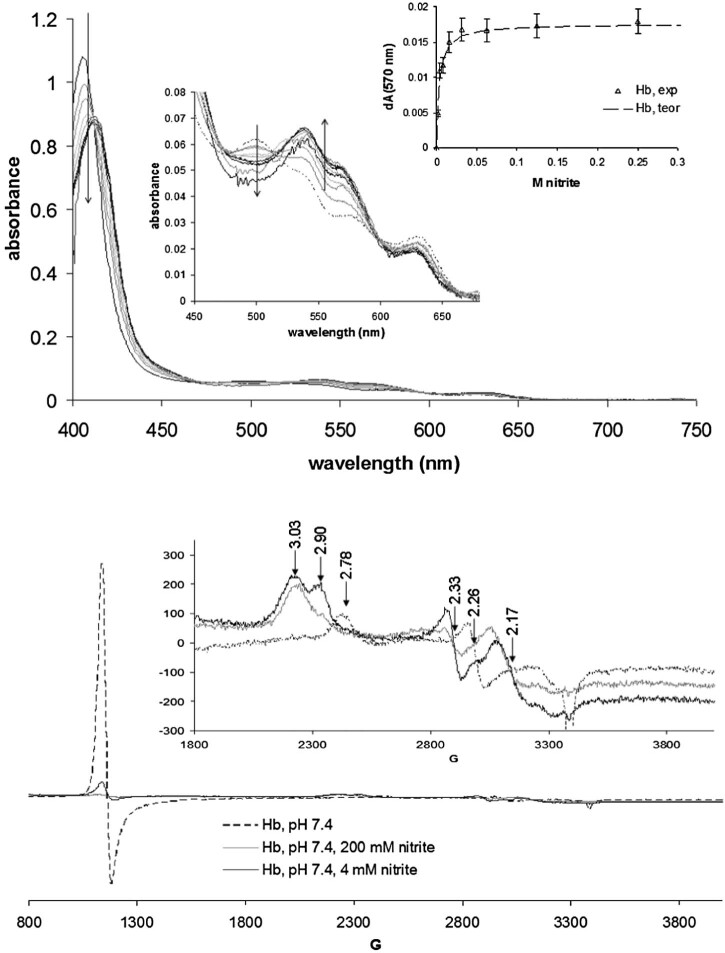
Top panel: UV-vis spectra of hemoglobin (100 µM) in the presence of varying amounts of nitrite; shown as inset is a binding curve whose theoretical fit indicates a binding constant of 3 mM. Bottom panel: EPR spectra of Hb in the presence and absence of nitrite, Conditions are as in Fig. 2.

UV-vis spectra were recorded on Agilent 8453 (Agilent, Inc.) and Cary 50 (Varian, Inc) instruments. EPR spectra were recorded at 10K in a Bruker EMX spectrometer, with a spherical high quality Bruker resonator SP9703 and an Oxford Instruments liquid helium system. The EPR measurements recorded at 100K was performed with a Bruker EMX EPR spectrometer equipped with a liquid nitrogen system (Supplementary materials).

A biologic SFM-300 UV-vis stopped-flow system equipped with a high-speed diode array detector was employed for stopped-flow measurements. Data were analyzed using the SPECFIT32 software package (BioLogic Science Instruments, Claix, France) using Singular Value Decomposition (SVD) and global multiexponential fitting of the SVD-treated data, with the spectra fitted to a simple A → B or complex A → B → C kinetic model and using Levenberg–Marquardt or Simplex algorithms.

Density functional calculations were performed following protocols previously validated [28], [29], [30], [31], [32]. Geometries were optimized for each spin state without any geometrical constraints, with the BP86 functional, which uses the gradient-corrected exchange functional proposed by Becke (1988) [33], the correlation functional by Perdew (1986) [34], and the DN** numerical basis set (comparable in size to 6-31G**), as implemented in Spartan [35]. For the SCF calculations, a fine grid was used, and the convergence criteria were set to 10^−6^ (for the root-mean square of electron density) and 10^−8^ (energy), respectively. For geometry optimization, convergence criteria were set to 0.001 au (maximum gradient criterion) and 0.0003 (maximum displacement criterion). Charges and spin densities were derived from Mulliken population analyses after DFT geometry optimization. Calculations were also performed in the Gaussian 09 package [36] with the same basis set and functional (for the larger models, this was essential due to convergence problems in the other software package).

**Fig. 4:**
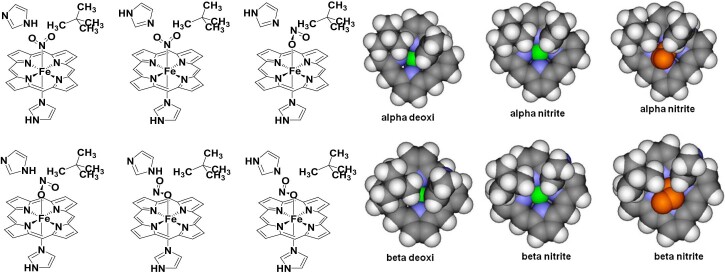
Left: extended models for the nitrite adducts of hemoglobin, taking into account sterical and hydrogen bonding conditions at the distal pocket around the nitrite. Right: views of the heme, perpendicular to the macrocyclic plane from a direction trans to the proximal histidine, obtained from crystal structures of Hb in the deoxy and in the ferric nitrite-bound forms (only the atoms included in our computational models are shown). These structures were employed as starting points in our calculations. Color code: carbon – gray, hydrogen – white, iron – green, porphyrin nitrogen – blue. The nitrite is shown in orange.

Basic models consisted of a laterally-unsubstituted heme, ligated axially by an imidazole ligand mimicking the proximal histidine, trans to a nitrite ligand bound to the iron with Fe-N distances as indicated in Fig. 1. Additionally, two sets of larger models were also constructed, starting from the α and β subunits of deoxy hemoglobin (pdb entry 2DN2). As indicated in Fig. 4, these contain the laterally-unsubstituted heme coordinated axially with protonated imidazole and nitrite respectively; to simulate the steric effect that the protein may impose, the imidazole ring of the distal histidine and the side-chain of valine 62 (cf. 2DN2 numbering) were included, and during the optimization all heavy atoms except for iron and nitrite were frozen. For each of these models both Fe(II) and Fe(III) oxidation states and low and high spin states were analyzed. Optimized structures were compared with the one described in literature for the Hb-nitrite adducts (pdb entry 3D7O).

**Table 1:** Partial occupancies (spin densities, i.e. α-β differences) for the iron d_xz_ and d_yz_ orbitals in models of the nitrite adducts of cytochrome cd_1_ nitrite reductase (at previously reported geometry and after rotation of the nitrite ligand by 45°, respectively) and of globins (‘Mb’ – freely optimized, ‘45°’ – nitrite ligand rotated around the Fe-N bond by 45°; ‘Mb OH^−^ – nitrite replaced with hydroxide; Mb CN^−^ – nitrite replaced by cyanide. Data from UBP86/6-31G** geometry optimizations followed by Mulliken population analyses.

	d_xz_	d_yz_	d_xz_/d_yz_
cd_1_ NO_2_^−^	0.39	0.32	1.2
cd_1_ NO_2_^−^ 45°	0.78	0.08	9.8
Mb NO_2_^−^	0.63	0.18	3.5
Mb OH^−^	0.63	0.08	7.9
Mb CN^−^	1.08	0.08	13.5
Mb NO_2_^−^ 45°	0.47	0.35	1.3

## 3 Results and discussion

### 3.1 Theoretical feasibility of in situ linkage isomerization

Fig. 1 shows a calculated potential energy surface for nitrite isomerization between the nitro and nitrito isomers in a simple heme model, ignoring any potential effects from surrounding protein/solvent. It appears beyond any doubt that the reaction barrier for such a process must be extremely low and hence both isomers should in principle be accessible kinetically, via isomerization starting from either end and without requiring the nitrite to fully dissociate in the process. This would be important in cases such as protein/enzyme active sites, where nitrite access in and out of the active site may be restricted. While it has been demonstrated on a number of occasions that the nitro and nitrito isomers of metalloenzyme active sites are very close in energy, including in globins [2], [31], [37], [38], this is the first report where a barrier for interconversion is computed and shown to be low.

### 3.2 UV-vis and EPR features of metmyogobin-nitrite adducts

Fig. 2 shows UV-vis and EPR spectra of ferric myoglobin in the presence of nitrite, at acidic and basic pH. The optical spectra indicate a binding constant of 14 mM for nitrite on Mb. The EPR spectra are thus recorded at full saturation (200 mM) and should only feature signals due to the nitrite-Mb adduct; it is thus seen that nitrite induces a low-spin signal which, due to its g~3 feature, is most likely attributable to a nitrogenous ligand [39]. This suggests that, contrary to what is seen in the crystal structure, in solution nitrite can in fact bind to the iron in myoglobin via its nitrogen atom. However, the area under this signal accounts for only 44% of the heme present in the sample [20]. A high-spin g~6 signal is also observed in the nitrite-treated sample, suggestive of a high-spin ferric state, accounting to 14% of the heme. In view of the optical titration data the aqua-like g~6 signal seen in the presence of 200 mM nitrite must be attributed to a nitrite adduct; due to the similarity in shape with the aqua signal, this nitrite g~6 form is most easily attributed as due to nitrite binding to iron via its oxygen atom. To our knowledge the g~6 signal in globins is largely unaffected by the identity of the axial ligand, so that it would not be unexpected for a nitrite adduct to feature a signal similar to that of the aqua.

**Table 2:** Distances (Å) and angles/dihedrals (°) illustrating the space available for nitrite binding at iron in the distal cavity. Data from crystal structures as detailed in the Experimental section. N(His) refers to the nitrogen atom closest to iron within the distal histidine; C(Val) refers to the carbon atom closest to the N(His) within the distal valine. N(heme) refer to the two heme nitrogens found trans to each other *and* closer to the N(His) and C(Val), respectively; Supporting material Fig. S3 illustrates these distances and angles.

	N(His)-C(Val)	N(His)-Fe	C(Val)-Fe	N(His)-Fe-C(Val)	N(His)-N(heme)-N(heme)-C(Val)
α nitrite	3.71	4.18	5.03	46.39	2.61
β nitrite	3.81	4.45	4.72	49.05	0.56
α deoxy	3.82	4.28	4.96	48.11	8.99
β deoxy	3.41	4.05	4.03	49.45	3.47

**Fig. 5:**
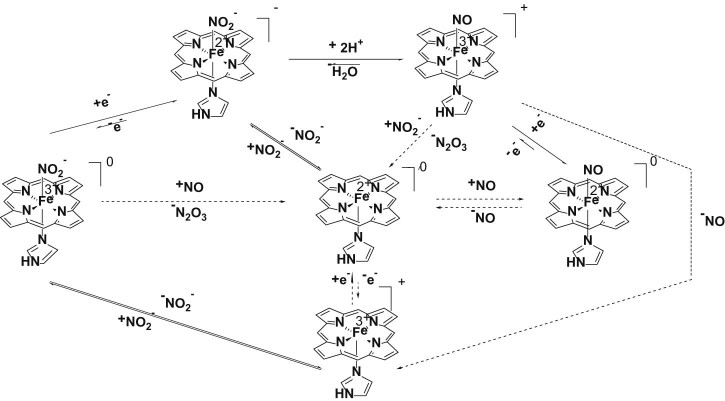
Schematic representation of the reactions that may occur at the treatment of ferric/ferrous hemoglobin with nitrite in the presence of a reducing agent. The dashed arrows mark reactions that may occur only after completion of one catalytic cycle.

Under the interpretation given below, both linkage isomers of Mb-nitrite must be present at the same time in solution – an experimental finding which to our knowledge is unprecedented in *metalloproteins*. An alternative interpretation, whereby the binding constant for nitrite is temperature-dependent and hence the g~6 signal observed at 10K is due to nitrite-free globin, is also feasible; this change in affinity would have to be more than one order in magnitude and might be under allosteric control [15], [21]. On the other hand, the accessibility of the o-isomer in globins at low temperatures is already proven by crystallographic data [17], [18]. The presence of high-spin and low-spin components of the nitrite adduct at room temperature is also supported by the UV-vis titration: even at saturating nitrite concentrations, a well-defined absorption maximum remains at ~625 nm, which is a hallmark of high-spin globins – while at the same time the increase in absorbance at 530–580 nm is indicative of low-spin species also being present.

### 3.3 UV-vis and EPR features of methemogobin-nitrite adducts

UV-vis and EPR spectra of the nitrite adduct of ferric human hemoglobin are shown in Fig. 3. The optical spectra suggest that Hb features a ~5-fold increase in affinity toward nitrite compared with Mb (3 mM vs. 14 mM); other hemoglobins (cow, horse, dog, rat, sheep – data not shown) likewise show similar affinities, and clear evidence of cooperativity was not found in any of them. The apparent EPR-silence observed in the previous reports [10] for the Hb-nitrate adduct was later interpreted as a saturation of the low spin species at a lower microwave power [15]. Our data are in agreement with others citing where the EPR signal due to the Hb-nitrate signal is partially visible with some dependence on the buffering agent. Although the signal is smaller than expected they are visible at g~6 and g~3 (similar to the Mb-nitrite adduct), suggesting that linkage isomerism is also at work in Hb. Furthermore, two sets of low-spin signals are seen for the nitrite adduct (3.03/2.33 and 2.90/2.17), which suggests that at least two possible distinct conformations are available for the Fe-NO_2_^−^ moiety; these two conformations are likely possible on the same subunit as opposed to being restricted on the α and β subunits, respectively, since the Fig. 2 Mb-nitrite EPR also shows some asymmetry in the g~3 signal and since there seems to be a concentration and pH dependence on their relative distributions (cf. Supporting material). Also, the experiments performed at 100°K, in concordance with the results obtained by Goetz et al. [15] (Supplementary material Fig. S1), show a direct correlation between the square root of the power applied and the intensity of the g = 6 for MetHb, as well as for MetHb-nitrate*.* Fig. 2, Fig. 3 also reveal a small difference between the electronic absorption UV-vis spectra of the Hb and Mb nitrite adducts, with the 530–580 nm features, specific to low-spin heme, being more pronounced in hemoglobin than in myoglobin. Interestingly, this difference is mirrored by the EPR spectra, where addition of nitrite causes the g~6 signal to decrease distinctly more in Hb than in Mb.

### 3.4 Origin of EPR silence in globin-nitrite adducts

The buffer-dependent EPR ‘silence’ of heme-nitrite adducts (in the N-ligated form) has previously been noted and several explanations put forth. One of these, based on uniaxial state considerations of Palmer, assumes that the single unpaired electron of the low-spin ferric–nitrite heme can be shared equally between the d_xz_ and d_yz_ orbitals of the metal (where the z-axis is defined perpendicular to the heme and along the Fe-N(nitrite bond) [20], [40]. Although we have previously reported geometries of the ferric-nitrite adduct of heme *d*_1_ (a model of the state experimentally known to be EPR-silent) [2], a detailed account of d-orbital occupancies was not given at that time. We now report in Table 1 that for this *cd*_1_ model the spin densities on the iron d_xz_ and d_yz_ orbitals, computed from Mulliken population analyses following DFT geometry optimization, are almost equal to each other in the ferric–nitrite heme *d*_1_ model and hence would fulfill Palmer's uniaxial [20] condition. Table 1 also shows that upon simple rotation of the nitrite ligand by 45° around the iron–nitrogen bond, the d_xz_/d_yz_ ratio increases dramatically, taking us outside the uniaxial condition. The same is true with a *b*-type heme, such as in myoglobin. Thus, a freely-optimized ferric–nitrite heme *b* model shows a d_xz_/d_yz_ ratio distinctly larger than 1, albeit smaller than what is seen when the nitrite is replaced by other ligands (hydroxide, cyanide). However, simple rotation of the nitrite ligand by 45° around the Fe–nitrite bond results in a drastic decrease of the d_xz_/d_yz_ ratio. We therefore conclude that difficulties in observing EPR spectra in heme nitrite complexes can indeed be adequately explained by Palmer's uniaxial state considerations [20]. Furthermore very subtle structural change, i.e. rotation around the Fe–nitrite bond, can dramatically affect the ratio of occupancies of d_xz_ and d_yz_ and hence the strength of the EPR signals detectable.

**Fig. 6:**
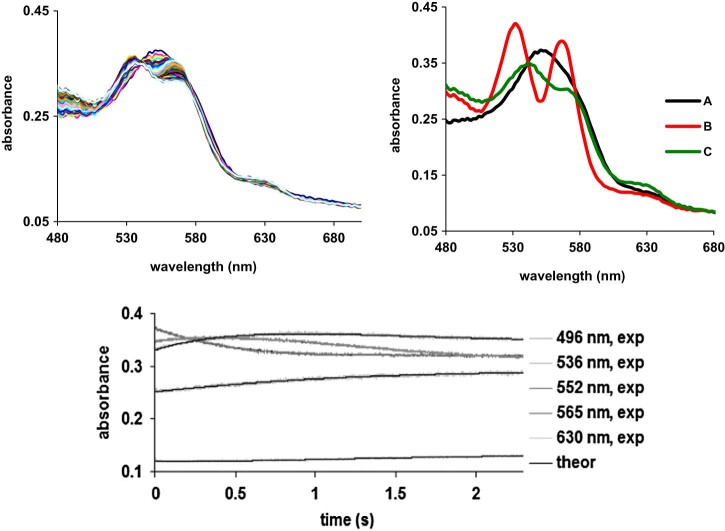
Upper panel: left UV-vis spectra of deoxyhemoglobin treated with nitrite, recorded for up to 2 seconds after mixing. Conditions: deoxyhemoglobin was obtained by purging with argon, and then adding a glucose oxidaze/glucose/catalase mixture as indicated in Experimental, buffer PBS, pH = 7.4, [Hb] = 18 µM, [nitrite] = 925 mM. Right: involved species as resulted from the fitted spectra, employing the A → B, B → C model. Lower panel: left: time evolution of the three fitted species, A, B and C; right: overlay of kinetic data and of fitted trace at some representative wavelength.

### 3.5 Differences between α and β chains of hemoglobin

The computations described in the previous section on the globin-nitrite adducts were extended to models including two distal aminoacids (the histidine and the isoleucine), observed in crystal structures to be within non-covalent contact with small ligands (cf. Fig. 4). These aminoacids are expected to modulate binding of nitrite, especially as their positions vary slightly form subunit to subunit, or indeed from globin to globin. Fig. 4 illustrates such differences: the iron is slightly more accessible in the α subunit, both in the deoxy and in the met-nitrite forms; moreover, a rearrangement of the distal aminoacids from the deoxy to the nitrite structure is more evident in the β than in the α subunit. Nevertheless, Table 2 shows that the distance between these two distal aminoacids, as well as the distance from the iron, does not change significantly between the deoxy and the met-nitrite states.

Attempts to model high-spin states for n-isomers failed for both subunits, due to sterical constraints: upon geometry optimization the Fe–N bond was broken, the nitrite eventually reorienting itself with an oxygen atom toward the iron. This was true for ferrous as well as ferric models, and suggests that indeed, as also suggested in the preceding sections, the high-spin signals in the EPR spectra of globin-nitrite adducts are likely due to the o-isomers.

Attempts to model the low-spin state of the n-isomer were also met with failure in some of the models, as the sterical constraints above the iron led to de-ligation of the nitrite. Nevertheless, as a general rule, these sterical constraints meant that the n-isomer was always found to be less stable than the o-isomer, by 10–35 kcal/mol depending on the model – in contrast to the data in Fig. 1 and thus offering an example of active site modulation for linkage isomerism. Interestingly, for the low-spin states, the d_xz_/d_yx_ ratio (as defined in Table 1) was computed to be 8.5 for the o-isomer (close to the value shown in Table 1 for a model of the well-observable ferric-hydroxo adduct) and only 2.1 for the n-isomer, suggesting that EPR visibility will differ between the n- and the o- isomers. Furthermore, as Table 1 indicates, in globin ferric–nitrite models the d_xz_/d_yx_ ratio varies from 1 to 3.5 within the span of only a 45°-rotation of the nitrite around the Fe–NO_2_ bond; even smaller changes in this angle would thus be likely to cause changes in this ratio and hence, within the framework of the uniaxial interpretation, control the degree to which this species would be EPR-detectable.

### 3.6 Reaction of deoxy hemoglobin with nitrite

Fig. 5 illustrates the reactions that may occur upon the treatment of ferric or ferrous hemoglobin with nitrite in the presence of a reducing agent. Ferric hemoglobin binds reversibly to the nitrite generating a complex that may undergo reduction in the presence of a reductive agent forming a ferrous hemoglobin – nitrite complex which, in our knowledge, is yet to be characterized – beyond a report concerning a UV-vis spectrum recorded on a crystal subjected to X-rays in reference [41]. This ferrous–nitrite complex may then, in a proton-dependent process, release a water molecule and form a ferric-NO adduct. The metHb-NO complex can be further reduced to Fe(II)-NO, react with nitric oxide and generate deoxyHb and N_2_O_3_, or liberate NO to form metHb. Rifkind and co-workers have described in some detail reactions occurring on longer time scales, of the order of minutes, where ferrous-nitrosyl adducts are indeed eventually detected, with proposed modulation by cysteine 93 [42], [43], [44], [45]. They have additionally postulated, based on quasi-steady-state data, that non-negligible amounts of ferrous-nitrite adduct are formed in their experiments.

Fig. 6 shows the UV-vis spectra collected during the reaction of deoxyhemoglobin with excess nitrite. The first spectrum, characterized by a band with a maximum at 552 nm, is attributable to the starting deoxyhemoglobin, while the last one, characterized by three absorption maxima at 502, 531 and 590 nm, is a mixture of the met-nitrite adduct with the Fe(II)-NO (Supporting material Fig. S2), findings in concordance with other studies [12]. The EPR spectrum of species C confirms the presence of the Fe(II)-NO adduct, even if the superhiperfine coupling is not completed resolved (Supporting material Fig. S3). The intermediate, species B in Fig. 6, bears excellent resemblance to the ferric-NO adduct. The implication is that, en route from deoxy to met-NO, the ferrous-nitrosyl is formed at a rate significantly slower than its decay, and that it, therefore, does not accumulate to a level detectable by UV-vis spectroscopy even under large excess of nitrite.

**Fig. 7:**
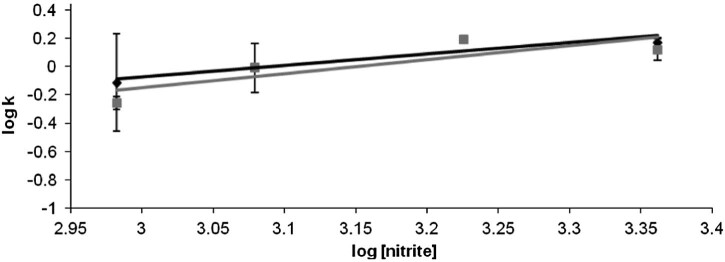
Dependences on nitrite concentration for the two steps of the deoxy → met nitrite process discussed in Fig. 6. The rate constants are 0.08 M^−1 ^s^−1^ for k_l_ (A → B, black symbols in Fig.), and 0.04 M^−1 ^s^−1^ for k_2_ (B → C, grey symbols); the reaction orders are 0.8 and 1, respectively. At nitrite concentrations below 400 mM, essentially no transformation of deoxy hemoglobin was observed under these conditions.

Fig. 7 illustrates the dependences on nitrite concentration for the two steps of the oxy → met nitrite process discussed in Fig. 6. In line with our interpretation of the data, both processes depend on this concentration; the rate constants for the two consecutive processes are very similar to each other.

To conclude, we have described here the binding of nitrite to ferrous and ferric hemoglobin. EPR evidence for linkage isomerism was presented, and a DFT-derived explanation given for the partial EPR silence of the globin ferric-nitrite adducts. Kinetic experiments targeting nitrite reduction by hemoglobin have failed to observe a ferrous-nitrite adduct.
