# Supplementary material for: Nitrite binding to globins: linkage isomerism, EPR silence and reductive chemistry
Source: Nitric Oxide. 2014 Nov 15;42:32–9. doi: 10.1016/j.niox.2014.08.007 (PMC4256065; doi:10.1016/j.niox.2014.08.007)
Supplement: Appendix S1 — Figs. S1–S3. [file mmc1.docx]

Nitrite binding to globins: linkage isomerism, EPR silence and reductive chemistry

Radu Silaghi-Dumitrescu,^a,b*^ Dimitri A. Svistunenko,^b^ Daniela Cioloboc,^a^ Cristina Bischin,^a^ Florina Scurtu,^a^ Chris E. Cooper^b^

^a^ “Babeş-Bolyai” University, 1 Mihail Kogalniceanu str., RO-400084 Cluj-Napoca, Romania, and ^b^ Department of Biological Sciences, University of Essex, Wivenhoe Park, Colchester, Essex, UK, CO4 3SQ

*to whom correspondence should be addressed, rsilaghi@chem.ubbcluj.ro


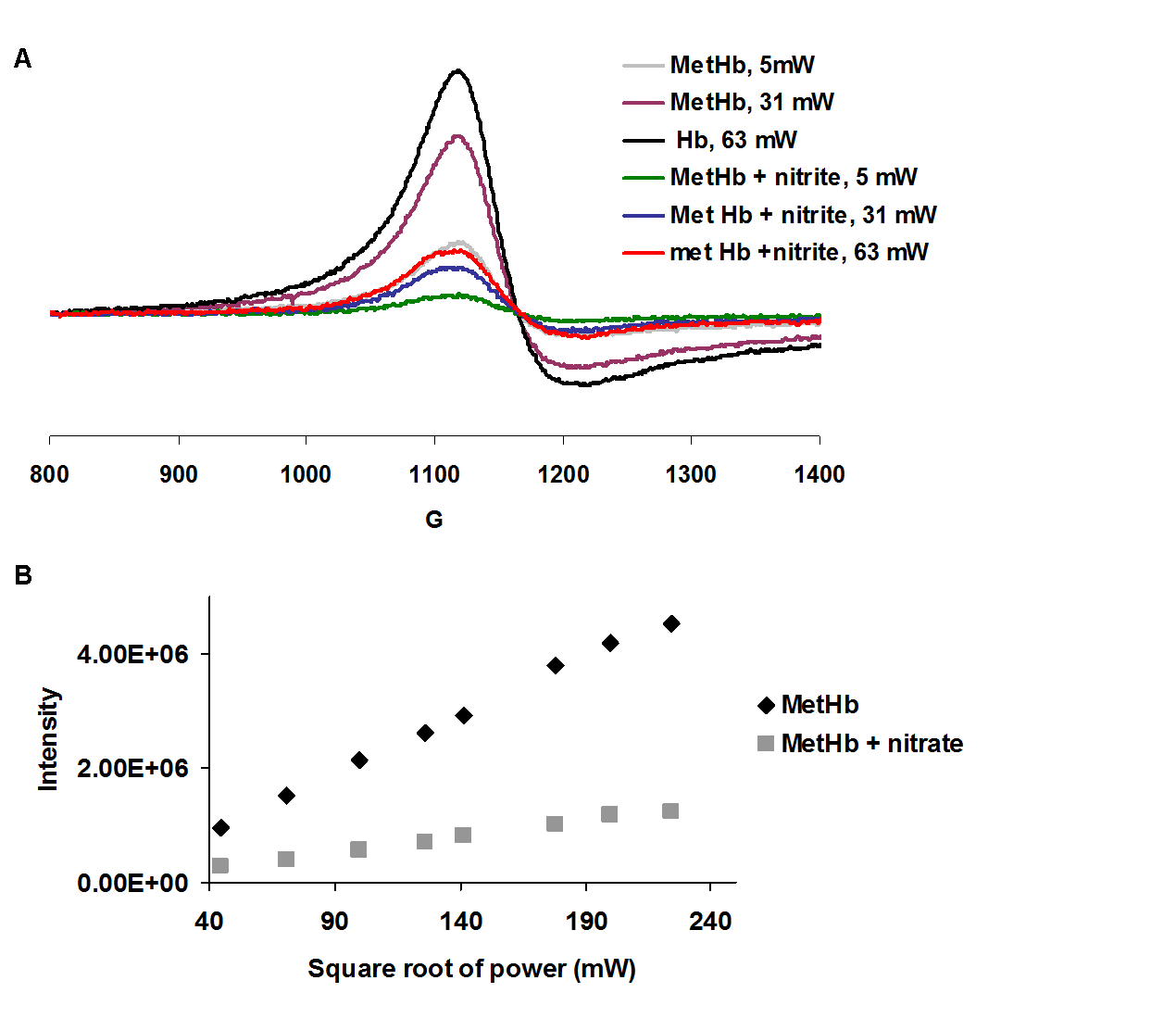


Fig. S1. A) EPR spectra of the high spin MetHb (100 µM) treated with nitrate (200 µM), in phosphate, pH=7,4, measured at 100°K, obtained at the microwave power indicated in the figure legend. B) EPR peak intensities of high spin MetHb in the presence/absence of nitrate using different microwaves power; conditions are the same as in the A.


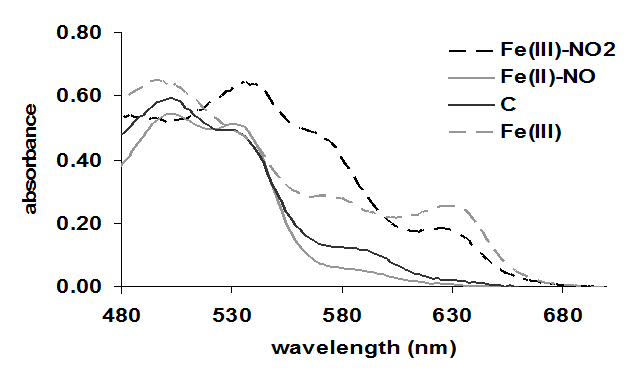


Fig. S2. Overlay of the computed spectra of species C with other possible species that are involve in the deoxy-Hb-nitrate reaction described in the figure legend.


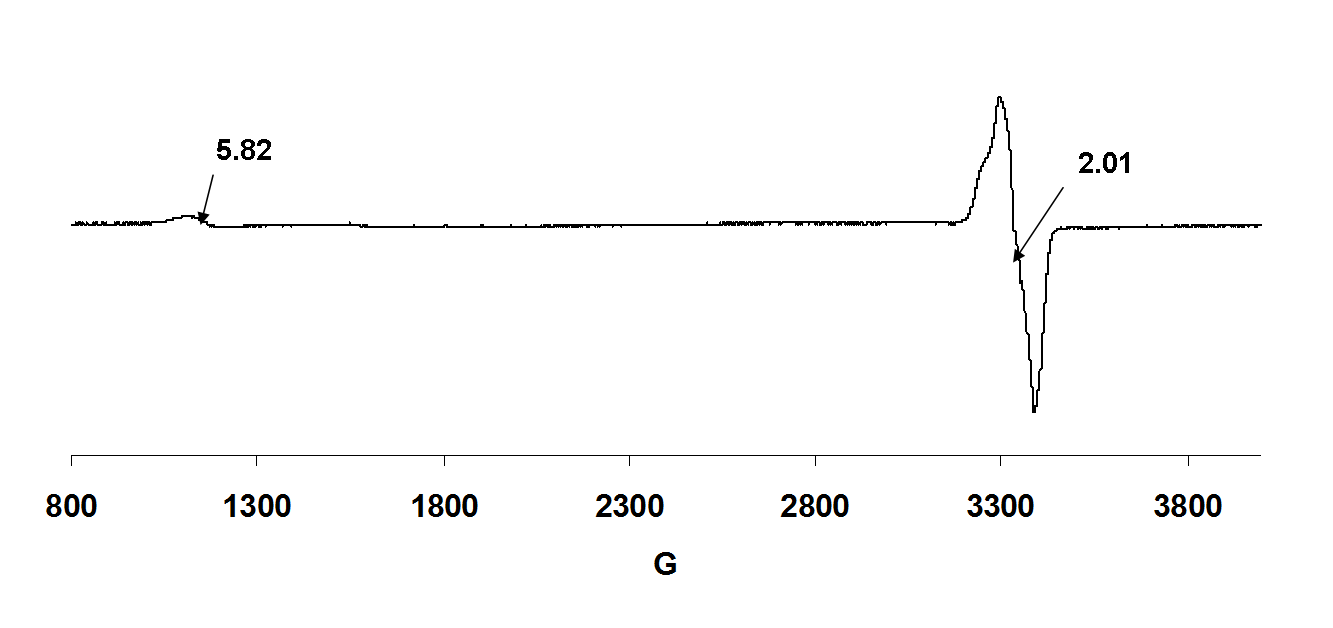


Fig. S3. The EPR spectrum of the proposed species C, obtained at 20 seconds after mixing of deoxy-Hb (50 µM) with nitrite (150 mM). Collected with a Bruker EMX EPR spectrometer with continuous wave at X-band (~9 GHz)
